# Typhoid Fever and Filtration

**Published:** 1907-04

**Authors:** 


### BUFFALO MEDICAL JOURNAL.

A Monthly Review of Medicine and Surgery.

*EDITOR:*

WILLIAM WARREN POTTER, M. D.

All communications, whether of a literary or business nature, books for review and exchanges, should be addressed to the editor 238 Delaware Ave., Buffalo, N. Y.

Vol. LXII. APRIL, 1907. No. 9

### Typhoid Fever and Filtration.

IT is an undisputed scientific fact that typhoid fever is a water borne disease in large part; but it is an equally undisputed fact that typhoid disease is communicated through other channels. For example, contaminated oysters have been charged with disseminating the disease, while milk is another medium through which the germs of the malady are distributed.

It is important, however, that the water supply of a large city should be placed above suspicion, since drinking water constitutes the greater medium of infection. Filtration is one of the methods offered to purify the water supply of Buffalo and if we may judge by the results at Albany, it ought to prove adequate. The experience at Washington, however, may be cited as somewhat unusual after the establishment of a filtration plant. This improvement took two years of time and cost $3,000,000 to construct, being finished in October, 1905. It met the approbation of sanitary experts and gave hope that the difficulty was removed. It was soon discovered, however, that the death rate from typhoid for a portion of the year following the establishment of the plant was as great as for a corresponding period in the previous year. This created a suspicion that the filters were imperfect or were not a safeguard against the spread of typhoid disease.

It was a serious indictment against filtration if true, and led the "Engineering News" to investigate the entire problem. Mr. Theodore Horton, consulting engineer of the New York State Department of Health, was engaged to go over in detail the conditions prevailing at Washington and his report is an interesting one for several reasons. He points with emphasis to the fact that drinking water is not the only medium for the dissemination of typhoid germs; he expresses the opinion that contaminated oysters have contributed to the infection at Washington; he thinks the milk supply, though rather closely watched, is not guarded sufficiently; that some of the water consumed in the city is taken from shallow wells; and, finally, he suggests that southern cities fare worse than northern cities in relation to typhoid disease and assigns Washington to the former class.

We have invited attention to this matter because we deem it important that every available light should be turned on the screen while the subject is under advisement in Buffalo. We do not cite the experience at Washington as a reason for discouragement so far as the establishment of a filtration plant is concerned, but rather to caution against a too sanguine expectation that typhoid fever will disappear entirely and immediately after such a plant is installed.

Whatever is done to improve the quality of our water supply should have the approval of sanitary experts and engineers of the first order. Nor must it be forgotten that there always remains in a large city what has been called "residual typhoid"; that is to say, a number of cases will always lurk in the nooks and crannies of a great city, sometimes even in its best residential quarters. A few such come from other cities and towns, through the incidents of travel or the fluctuation of population.

It is not unreasonable to indulge the hope, in spite of all the difficulties in the way, that some method will be evolved which will give to Buffalo the most complete, adequate, and sanitary water supply yet instituted in a populous city.

---

But while Buffalo is striving to put its water supply on a high sanitary plane, she must not forget the injustice of contaminating waters that go to neighboring cities or towns. To dump the sewage of this large and ever growing city, now numbering, conservatively speaking, 400,000 souls, into the Niagara river and thus contaminate the source from which Tonawanda and Niagara Falls must needs take their water supplies, is not only an injustice but comes very near being a crime. Now that the Passaic river, whose waters have so long been befouled between Paterson and Newark, is about to be cleansed by withholding the sewage heretofore dumped into it by the town and cities along its borders, especially Paterson, is it not high time for Buffalo to clear its own skirts of the charge of increasing the typhoid rates of its neighbors downstream?

---

It is not pleasant to scold one's own city for its shortcomings but it is only fair that these should be pointed out, now and then, lest they be overlooked to its disadvantage in comparison with its neighbors of the same class. In the controversy growing out of repeated attempts to establish a union railway station, the rivalry which is almost a quarrel between the east and west sides, has permitted us to continue a disgrace, so far as our railway stations are concerned, that has become almost intolerable. Buffalo, too, permitted itself to be inveigled, if not swindled, into establishing its postoffice site in a side street in the midst of an environment that is disgraceful, to say the least.

Cleveland, our sister city, on the other hand, with far less lake and railway advantages, locates its government building, its municipal offices and its great railway station on a plaza in the very center of its best locality. The buildings, too, represent the very highest type of architectural beauty and usefulness. Is it any wonder, then, that Cleveland has outstripped Buffalo in its growth, not only of population but of financial prosperity and its growth, not only of population but of financial prosperity, and in its cultivation of the arts and sciences as well as in the esthetics of civic standards? And this, too, with lesser commercial advantages and of inferior railway facilities!

---

Dr. Ernest Wende, health commissioner, is leading a crusade against pipeless stoves and it is reported that Corporation Counsel Desbecker is now preparing an ordinance prohibiting the sale or use of gas stoves which do not provide for connection with a flue. The recent death of two women, sisters, from gas asphyxia, has awakened interest in this subject and we trust the common council will not be slow in approving the ordinance in question. It is distinctly a measure, humane and necessary, to prevent a recurrence of deaths from such a cause.

---

The "Buffalo Express," in its edition of March 13, 1907, says: "The man who spits on a sidewalk is a swine. If public opinion in Buffalo would not support the enforcement of an ordinance forbidding such a filthy offense, shame on Buffalo!"

Dr. William C. Callanan, alderman of the twentieth ward, offered a resolution in the aldermanic board recently, extending to public walks the ordinance that now prohibits spitting in street cars and public buildings. No opposition should be made to this measure, it being distinctly in line with approved reform in municipal sanitation.

---

Dr. Herbert Claiborne, of New York, says the Philadelphia Record, suffers from cold hands in winter. And nothing will warm his fingers except hot water, a hot fire or a hot potato. He can be seen almost any frosty morning marching along at five miles an hour with a hot potato in each overcoat pocket and his hands grasping the two big potatoes, piping hot, wrapped in silk handkerchiefs, for this purpose. "They will keep your hands warm for hours unless you happen to sit on 'em," he says. "They are great for a football match or when you go sleighriding."

